# Multimodal Management of Morbihan Disease: Isotretinoin, Intralesional Triamcinolone, and Ketotifen in a Recalcitrant Case

**DOI:** 10.7759/cureus.97314

**Published:** 2025-11-20

**Authors:** Brian A Moreno, Juan Rodriguez-Puerto, Kristin Haushalter

**Affiliations:** 1 Dermatology, Lake Erie College of Osteopathic Medicine, Bradenton, USA; 2 Dermatology, University of Miami, Miami, USA; 3 Dermatology, Derm360, Miami, USA

**Keywords:** dermatology care, dermatology case report, dermatology consult, dermatology outpatients, dermatology screening, dermatology teaching, dermatology treatments, general dermatology, research in dermatology, skin disease/ dermatology

## Abstract

Morbihan disease, also known as rosacea-associated solid facial edema, is a rare and chronic condition that presents with persistent facial swelling and often proves difficult to treat. We describe the case of a 57-year-old man with a four-year history of right-sided facial edema who was diagnosed with Morbihan disease after extensive prior evaluations. Intralesional triamcinolone injections at a tertiary center produced his best response, and combination therapy with isotretinoin was recommended. At our clinic, isotretinoin was initiated at 20 mg daily and titrated over several months, with doses adjusted between 10 and 40 mg based on efficacy and tolerability. A reduction to 10 mg coincided with worsening edema, while escalation improved symptoms. Adjunctive therapies included topical roflumilast, oral ketotifen, and repeat intralesional triamcinolone. Serial laboratory monitoring revealed persistent hyperlipidemia, though liver and renal function remained normal, necessitating primary care referral for lipid management. The patient reported partial improvement, most notably with higher isotretinoin dosing and intralesional corticosteroid injections, though intermittent flares persisted. This case highlights the challenges of treating Morbihan disease, emphasizing the role of individualized isotretinoin dosing, intralesional triamcinolone as a useful adjunct, and the importance of close safety monitoring and patient-centered decision-making in managing this rare condition.

## Introduction

Morbihan disease, also referred to as rosacea-associated solid facial edema, is a rare and chronic condition characterized by persistent, non-pitting swelling of the upper and middle face. It is considered a rare entity, with relatively few cases reported, which contributes to diagnostic uncertainty and delayed recognition. The disease often develops insidiously and can lead to significant cosmetic and functional impairment. Its pathogenesis remains incompletely understood, though proposed mechanisms include vascular dysregulation, lymphatic obstruction, and chronic inflammation associated with rosacea [1-3]. Histopathologic findings are variable, frequently showing dermal edema, dilated lymphatics, and perivascular lymphohistiocytic infiltrates, with some reports noting mast cell prominence [1,4].

Diagnosis is challenging due to clinical overlap with conditions such as angioedema, lupus erythematosus, dermatomyositis, and Melkersson-Rosenthal syndrome [1,2]. As a result, many patients undergo prolonged diagnostic journeys and are seen by multiple specialists before receiving a definitive diagnosis [2,3].

Therapeutic options are diverse but frequently unsatisfactory. Oral isotretinoin has demonstrated efficacy in several case series and systematic reviews, particularly when used in combination with intralesional corticosteroids [3,4]. Other reported treatments include systemic antibiotics such as doxycycline [5], oral corticosteroids [6], intralesional triamcinolone [4], and immunomodulators such as tofacitinib [7]. Non-pharmacologic approaches, including complete decongestive therapy [8] and surgical debulking [9], have also been described in refractory cases. Despite this range of interventions, relapse is common, and no standardized treatment algorithm exists.

We present the case of a 57-year-old man with a four-year history of Morbihan disease who demonstrated partial but clinically meaningful improvement with a multimodal regimen including isotretinoin, intralesional triamcinolone, and adjunctive therapies. This case underscores the complexity of management and highlights the importance of individualized treatment strategies in this rare disorder. In addition, this case illustrates a multimodal approach incorporating isotretinoin, intralesional triamcinolone, and ketotifen, and underscores a dose-dependent response to isotretinoin requiring careful titration and monitoring.

## Case presentation

**Figure 1:**
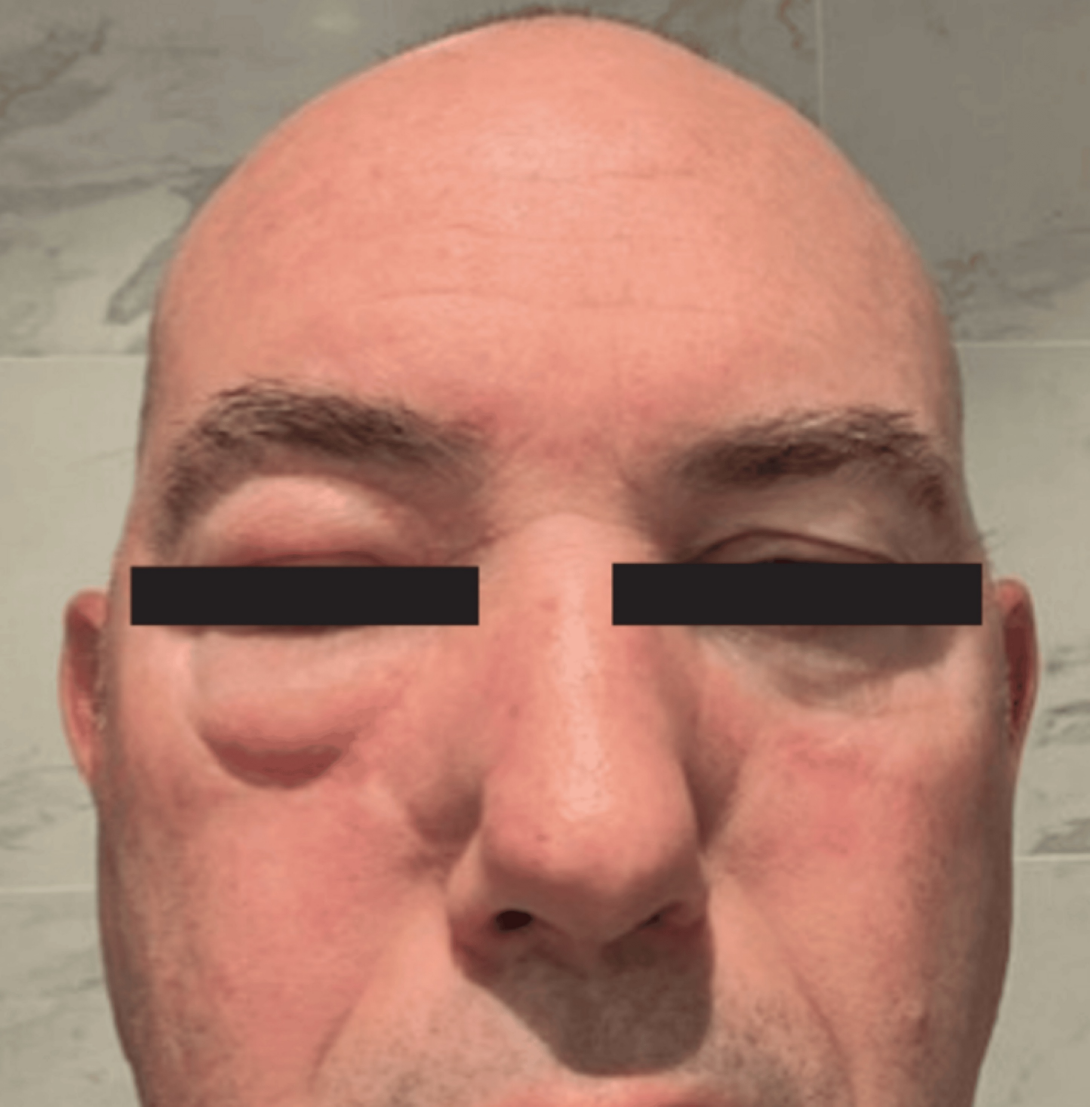
Chronic, non-pitting swelling of the right side of the face.

A 57-year-old man presented with a four-year history of chronic, non-pitting swelling of the right side of his face (Figure 1). He had previously been evaluated by more than 20 clinicians before receiving a diagnosis of Morbihan disease at the Mayo Clinic, where intralesional triamcinolone (ILK) injections produced his most significant improvement to date. The diagnosis of Morbihan disease was established at an outside tertiary center based on the chronic, non-pitting solid facial edema and exclusion of alternative causes, per patient report. According to the patient, evaluations at that time ruled out other etiologies such as angioedema, connective tissue diseases, and Melkersson-Rosenthal syndrome. Original records from these evaluations, including any biopsy or imaging findings, were not available for review at our institution, which represents a limitation of this report. On presentation to our clinic, examination revealed firm, non-tender, non-pitting edema with erythema involving the right cheek, periorbital region, temple, and eyebrow area, without nodules, warmth, or systemic or ocular symptoms.

Isotretinoin was started at 20 mg daily. After one month, partial improvement was noted, and the dose was increased to 30 mg daily. Laboratory studies performed at that visit showed elevated cholesterol and triglycerides, which prompted repeat fasting evaluation. With continued, though incomplete, clinical improvement, the isotretinoin dose was increased further to 40 mg daily. The patient reported skin dryness as the main side effect and denied mood changes, gastrointestinal upset, or musculoskeletal symptoms.

After another month, the isotretinoin dose was reduced to 30 mg daily due to overall progress. At that time, topical roflumilast 0.3% foam was added to address concurrent seborrheic dermatitis and erythema. Several weeks later, the patient’s edema and erythema were further improved, and isotretinoin was decreased to 20 mg daily. Following another month of stability, the dose was reduced to 10 mg daily; however, the patient subsequently experienced recurrence of edema. He also received a short course of oral prednisone for a pulmonary indication during this time, which he felt subjectively improved his facial swelling.

At the next follow-up, the patient’s condition had worsened, and isotretinoin was increased back to 20 mg daily. Oral ketotifen 1 mg nightly was added, based on reports of adjunctive benefit with antihistamines. Laboratory monitoring demonstrated persistent hyperlipidemia with triglycerides ranging from 152-313 mg/dL and low-density lipoprotein (LDL) cholesterol up to 180 mg/dL, though liver and renal function remained normal. Primary care follow-up for lipid management was arranged. A summary of the available biochemical parameters during treatment is provided in Table 1.

**Table 1:** Summary of available biochemical trends during isotretinoin therapy. LDL, low-density lipoprotein; AST, aspartate aminotransferase; ALT, alanine aminotransferase

Parameter	Observed values	Interpretation
Triglycerides	152–313 mg/dL	Persistent elevation; monitored closely
LDL cholesterol	Up to 180 mg/dL	Elevated; prompted primary care referral
AST/ALT	Within normal limits	No hepatic toxicity observed
BUN/Creatinine	Within normal limits	Stable renal function

At a subsequent visit, the patient continued to experience persistent inflammation despite isotretinoin. Intralesional triamcinolone (2.5%, total 1 cc) was administered to the right cheek, temple, and periorbital region. Several weeks later, he reported limited improvement, and repeat ILK injections (2.5%, total 4 cc) were performed across multiple right facial sites, including the upper and lower eyelids, eyebrow, cheek, and temple.

**Figure 2:**
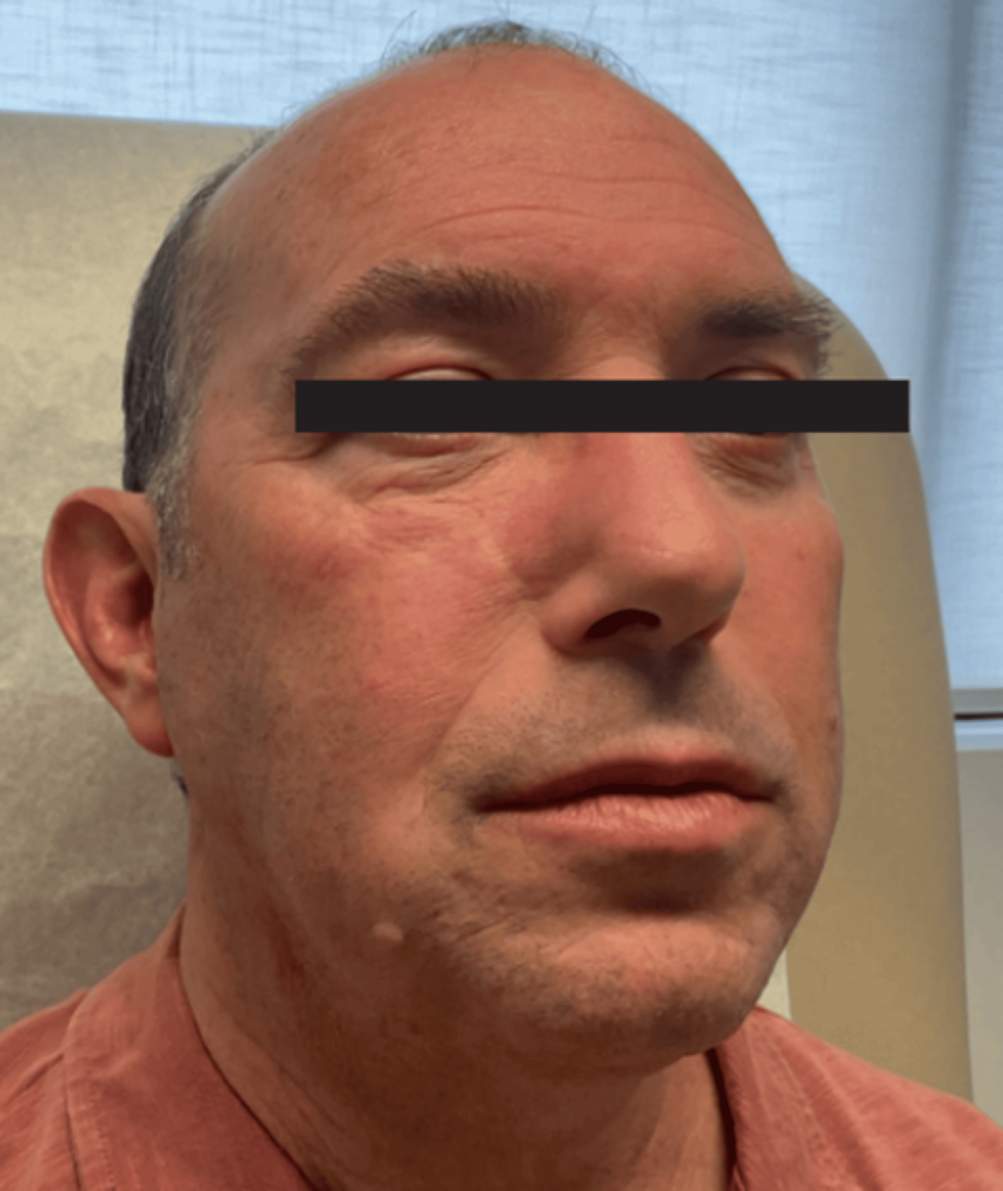
Most recent visit with persistent though somewhat attenuated swelling and erythema.

At his most recent follow-up, the patient remained on isotretinoin 40 mg daily, with persistent though somewhat attenuated swelling and erythema (Figure 2). He continued adjunctive roflumilast foam and ketotifen. He expressed a preference against systemic JAK inhibitors due to concerns about immunosuppression. The treatment plan included ongoing isotretinoin, repeat ILK as needed, lifestyle modification for dyslipidemia, and close monitoring in coordination with his primary care provider. Written informed consent was obtained from the patient for publication of this case report, and a signed consent form has been submitted to the journal.

## Discussion

Morbihan disease is a rare, chronic condition characterized by persistent, non-pitting facial edema, most often associated with rosacea. Its pathogenesis remains incompletely understood, though proposed mechanisms include lymphatic dysfunction, vascular dysregulation, and chronic inflammation [1-3]. Because of its rarity and nonspecific histopathologic findings, patients frequently experience delays in diagnosis and prolonged disease courses, as illustrated by this patient who was seen by numerous clinicians before receiving a definitive diagnosis at a tertiary center [2,3]. In our case, the initial diagnostic workup and histopathologic findings were performed at an outside institution and were not accessible for independent review, which we recognize as a limitation. As such, our report is limited by reliance on the patient’s account of prior diagnostic evaluation, and histopathologic confirmation could not be independently verified.

Management of Morbihan disease is challenging, with no standardized treatment algorithm and variable responses reported across studies [1,3]. Oral isotretinoin has shown the most consistent benefit in case series and systematic reviews, particularly when used at low to moderate doses over extended periods [3,8]. While many reports describe benefit with low to moderate dosing over prolonged courses, our patient’s course suggested a dose-dependent effect, with improved control at higher doses and relapse following dose reduction. Our patient’s clinical course was consistent with this pattern: he demonstrated improvement on isotretinoin, particularly at higher doses, with worsening noted when the dose was tapered. This dose-responsive relationship highlights the importance of individualized titration, as underdosing may lead to relapse while higher doses must be balanced against tolerability and metabolic side effects. In this case, repeated elevations in triglycerides and LDL cholesterol necessitated close monitoring and primary care coordination, reflecting the safety considerations emphasized in the literature [8].

Adjunctive therapies are frequently required. Intralesional corticosteroids, particularly triamcinolone, have been reported as effective in reducing localized edema, possibly through their impact on mast cell-mediated inflammation [4]. Our patient achieved meaningful but temporary improvement after multiple sessions of intralesional triamcinolone, supporting its role as a useful adjuvant in combination with isotretinoin. Additional therapies described in the literature include doxycycline, which has been reported to be effective in several cases, either alone or in combination with corticosteroids [5,6]. Other immunomodulatory approaches, such as tofacitinib, have demonstrated success in isolated reports [7], although patients may decline such options due to concerns about systemic immunosuppression, as in this case.

Non-pharmacologic strategies have also been reported, including complete decongestive therapy, which improved facial edema in two cases [8], and surgical debulking or lymphatic surgery in refractory disease [9, 10]. While these approaches were not pursued in this patient, they remain considerations for future management if pharmacologic interventions do not achieve adequate control.

This case illustrates several important points in the management of Morbihan disease. First, it highlights a multimodal regimen combining isotretinoin, intralesional triamcinolone, and ketotifen, which provided sustained partial improvement in a recalcitrant case. Second, it demonstrates a dose-dependent isotretinoin response, emphasizing the need for gradual titration and caution when tapering to avoid relapse. Third, it underscores the importance of routine lipid monitoring and coordination with primary care when prolonged isotretinoin therapy is required.

## Conclusions

Morbihan disease remains a therapeutic challenge due to its chronicity, rarity, and lack of standardized guidelines. This case supports the use of a multimodal approach incorporating isotretinoin with intralesional triamcinolone and adjunctive agents such as ketotifen. Our patient’s dose-dependent response highlights the importance of gradual isotretinoin titration and close metabolic monitoring to reduce relapse risk and ensure safety. Even when prior diagnostic records are unavailable, transparent acknowledgment of this limitation and careful, individualized management can provide clinically meaningful improvement.
